# Bilateral Upper and Lower Limb Complex Regional Pain Syndrome (CRPS) in a Lung Cancer Survivor: Diagnostic and Interventional Challenges in the Setting of Radiculopathy and Prior Radiotherapy

**DOI:** 10.7759/cureus.96683

**Published:** 2025-11-12

**Authors:** Tim Hsu, Shivang Patel, Jake Belli, Zachary Siegel, Kamal T Patel

**Affiliations:** 1 College of Medicine, Lake Erie College of Osteopathic Medicine, Bradenton, USA; 2 Anesthesiology, Lake Erie College of Osteopathic Medicine, Bradenton, USA; 3 Physical Medicine and Rehabilitation, Walter Reed National Military Medical Center, Bethesda, USA; 4 Pain Management, NeuSpine Institute, Wesley Chapel, USA

**Keywords:** budapest criteria, chronic neuropathic pain, chronic radiculopathy, complex regional pain syndrome type 1, radiation-induced brachial plexopathy

## Abstract

We present the case of a 64-year-old man with an unusual and diagnostically challenging presentation of Complex Regional Pain Syndrome (CRPS) Type I involving both the left upper and lower extremities. His illness was precipitated by an on-the-job injury and was further complicated by a prior history of adenocarcinoma of the lung treated with radiation, resulting in severe left upper extremity (LUE) weakness along with cervical and lumbar radiculopathy. The overlapping clinical picture of CRPS, lumbar and cervical radiculopathy, and presumed radiation-induced brachial plexopathy (RIBP) posed a significant diagnostic challenge. This case highlights the usefulness of applying systematic diagnostic criteria, such as the Budapest Criteria, in diagnosing CRPS, particularly in the setting of coexisting neurologic pathology. It further underscores the importance of early recognition, multidisciplinary evaluation, and individualized treatment planning in patients with non-classical presentations of CRPS, especially within the post-cancer cohort.

## Introduction

Complex regional pain syndrome (CRPS) is characterized by pain that is disproportionate to the intensity of a triggering factor. It is known to have motor, sensory, autonomic, and trophic manifestations but typically lacks a clear peripheral nerve or dermatomal pattern of distribution [1]. Symptom onset may be delayed following the initial injury. The clinical course of CRPS can range from mild, self-limiting symptoms to chronic, persistent pain that causes significant disability for the patient.

CRPS can be categorized into three forms. Type I (formerly reflex sympathetic dystrophy) occurs without an identifiable nerve injury, while Type II (causalgia) occurs in the presence of a defined nerve injury [1]. A third form has been described for patients who partially meet the criteria for either Type I or Type II but lack another explanation for their symptoms [1]. While this third category is less commonly recognized, its inclusion reflects ongoing efforts to improve diagnostic precision. The estimated overall incidence of CRPS is 26.2 per 100,000 person-years [1].

The initial presentation usually involves unilateral, disproportionate pain, hyperesthesia, allodynia, skin color changes, and swelling, more commonly affecting the upper limbs. Due to its wide array of symptoms and reliance on clinical diagnosis rather than definitive diagnostic tests, CRPS is often mistaken for various conditions, including peripheral neuropathy, cellulitis, or functional neurological symptom disorder [2]. This allows for further progression of CRPS in the absence of an accurate diagnosis, which subsequently hinders the effectiveness of noninvasive treatment options.

The diagnosis of CRPS remains clinical and is most often based on the Budapest Criteria, after exclusion of alternative causes [2]. Standardized diagnostic criteria aim to improve clinical communication and facilitate research.

Here, we present a complex case of a 64-year-old man with trauma-induced CRPS involving both the upper and lower extremities, accompanied by cervical and lumbar radiculopathy and suspected radiation-induced left upper extremity weakness, illustrating the diagnostic and therapeutic challenges of overlapping neurologic syndromes.

## Case presentation

This case discusses a 64-year-old male truck driver who presented to the pain clinic on April 17, 2025, with chronic neck and low back pain accompanied by progressive weakness, sensory disturbances, and functional impairment of the left upper extremity (LUE) and left lower extremity (LLE). The patient sustained trauma when he was struck from behind by the front loader of a truck on September 16, 2023. The following findings reflect his current pain clinic examination on April 17, 2025, and not the initial post-injury assessment. On examination, there was severe, sharp, stabbing neck pain radiating to the LUE and moderate low back pain radiating to the LLE. The patient exhibited vasomotor changes (temperature and color asymmetry), sensory abnormalities (allodynia and hyperesthesia), sudomotor dysfunction (edema and sweating changes), and motor/trophic alterations (weakness, dystonia, reduced range of motion) in both the left upper and lower extremities. Gait was disturbed, and the patient required a cane for ambulation.

This patient meets the Budapest Criteria for CRPS Type I based on both history and examination findings [1]. He reported ongoing, severe pain in the left upper and lower extremities that was disproportionate to his original workplace injury. He exhibited symptoms in all four diagnostic categories: sensory abnormalities (allodynia and hyperesthesia), vasomotor symptoms (temperature and color asymmetry), sudomotor dysfunction (edema and sweating), and motor/trophic changes (weakness, dystonia, and reduced range of motion). On physical examination, signs were present in multiple categories, including swelling and discoloration of the affected limbs, limited range of motion, and the need for an assistive device for ambulation. While concurrent cervical and lumbar radiculopathy was present, the pattern and extent of his symptoms, including autonomic and trophic changes, were not fully explained by radiculopathy alone, supporting the diagnosis of CRPS Type I per the Budapest Criteria.

The patient was diagnosed with cervical radiculopathy based on the presence of neck pain radiating to the LUE accompanied by numbness, tingling, and burning sensations, which are characteristic of nerve root compression. Lumbar radiculopathy was also diagnosed due to low back pain radiating to the left lower leg, with similar nerve root compression symptoms as in the upper extremity. A positive straight leg raise test further supported the lumbar radiculopathy diagnosis.

Electrodiagnostic studies demonstrated chronic, moderate left C5-C7 radiculopathies with axonal loss of the left ulnar motor nerve and reduced conduction velocity (39 m/s; normal > 50 m/s). There was mild-to-moderate sensorimotor neuropathy of the median and tibial nerves bilaterally, with prolonged distal latencies (e.g., left median 3.8 ms; right tibial 55.3 ms). Sensory responses were absent in several nerves, including the left dorsal cutaneous, sural, and saphenous nerves. F-wave latency was prolonged in both tibial nerves (left 59.2 ms). These findings are consistent with a mixed picture of polyneuropathy and multilevel radiculopathy.

MRI of the cervical spine revealed multilevel degenerative disc disease with foraminal narrowing, most pronounced at C5-C7, correlating with the electrophysiologic findings of chronic cervical radiculopathy. MRI of the lumbar spine demonstrated degenerative changes at L5-S1 with mild-to-moderate neural foraminal stenosis. Together, these imaging and electrodiagnostic findings support the diagnoses of both cervical and lumbar radiculopathies. A summary of the key electrophysiologic findings, including abnormalities consistent with cervical and lumbar radiculopathy and polyneuropathy, is presented in Table 1.

**Table 1:** Summary of EMG/NCS findings demonstrating cervical and lumbar radiculopathy with polyneuropathy. mV: millivolts; µV: microvolts; EMG: Electromyography; NCS: Nerve Conduction Study.

Nerve / Root	Abnormality	Latency (ms)	Velocity (m/s)	Amplitude	Interpretation
Left Ulnar Motor	Reduced velocity	-	39	5.1 mV	Axonal loss
Left Median Sensory	Prolonged distal peak latency	3.8	37	15 µV	Sensory neuropathy
Left Tibial Motor	Prolonged F-wave latency	59.2	34	8.4 mV	Axonal neuropathy
Left C5-C7 Roots	Chronic denervation, reduced recruitment	-	-	-	Chronic radiculopathy
Sensory (sural, dorsal cutaneous, saphenous)	Absent response	-	-	-	Sensory polyneuropathy

The patient has a history of prior thoracic radiation therapy, although the exact treatment field and timing were not available. Clinically, the patient developed weakness involving the left deltoid muscle, raising the possibility of radiation-induced brachial plexopathy (RIBP). However, given the lack of brachial plexus-specific electrodiagnostic testing or dedicated imaging, this diagnosis remains suspected rather than confirmed. The clinical and electrophysiologic findings were also consistent with C5-C7 radiculopathy, and these overlapping features make it difficult to determine the exact contribution of prior radiation exposure.

Prior treatments included physical therapy and a cervical epidural steroid injection, which provided only transient relief. His pain persisted despite a multidrug regimen that included gabapentin, duloxetine, tizanidine, tramadol, and muscle relaxants. Given his persistent and debilitating pain despite these measures, the patient underwent implantation of a spinal cord stimulator (SCS). At his two-week follow-up, he reported approximately 70% pain relief in his neck and lower back, with marked improvement in mobility and daily activities. He was able to return to his baseline functional level, including walking without assistance. At his two-month follow-up, the patient continued to experience similar pain relief and functional gains, with decreased reliance on analgesic medications.

## Discussion

CRPS presents a significant clinical challenge due to its complex pathophysiology and variable response to treatment. Early recognition of the disease process is crucial for non-invasive treatment options to be effective. The Budapest Criteria are currently the most widely accepted diagnostic criteria for CRPS due to their high sensitivity (99%) and higher specificity (68%) compared to the Veldman criteria [3]. The International Association for the Study of Pain now defines the Budapest Criteria as continuous pain that is disproportionate to the initial injury, along with fulfilling criteria for sensory, vasomotor, sudomotor/edema, and motor/trophic symptoms. The Budapest Criteria, which remain the most widely accepted diagnostic standard for CRPS, are summarized in Table 2. Our patient meets the Budapest Criteria for CRPS Type I, with severe, disproportionate pain and symptoms across all four diagnostic categories that cannot be fully explained by his concurrent cervical and lumbar radiculopathy.

**Table 2:** Budapest Criteria for diagnosing complex regional pain syndrome. Source: This table has been adapted from Reference [1].

Serial No.	Criterion	Symptoms / Examples
1	Continuing pain, disproportionate to any inciting event	-
2	At least one symptom in three of the four categories	Sensory: Hyperesthesia Vasomotor: Temperature asymmetry, skin color changes, skin color asymmetry Sudomotor/Edema: Edema, sweating, sweating asymmetry Motor/Trophic: Decreased range of motion, motor dysfunction, trophic changes
3	At least one sign in two or more categories during evaluation	Sensory: Hyperalgesia to pinprick, allodynia to light touch or temperature Vasomotor: Temperature asymmetry, skin color changes, skin color asymmetry Sudomotor/Edema: Edema, sweating changes, sweating asymmetry Motor/Trophic: Decreased range of motion, motor dysfunction, trophic changes
4	No other diagnosis better explains the signs and symptoms	-

While the pathogenesis of CRPS is not completely understood, the hallmark of the initial phase is characterized by an exaggerated inflammatory response to the inciting trauma [4]. Pro-inflammatory cytokines such as IL-1β, IL-6, and TNF-α play an important role in the pathogenesis, but recent literature attributes the flares of CRPS to calcitonin gene-related peptide (CGRP) and the edema to substance P [5].

CRPS typically begins in a single limb but can later spread to involve multiple limbs. In one study that followed 159 patients with CRPS from 2009 to 2020, only one patient (0.7%) presented with bilateral limb involvement [6]. In another study that followed 185 CRPS patients from 1998 to 2004, approximately 88% of patients initially presented with involvement of a single limb with progression to subsequent limbs, whereas only 12% of patients presented with involvement of two or more limbs at onset [7]. As the condition progresses, the most commonly observed pattern of spread is contiguous spread, characterized by a gradual and significant enlargement of the initially affected area [8]. In patients who develop spread to a second limb, the most common mechanism of spread is from arm to contralateral arm or leg to contralateral leg. Pain spreading from the arm to the ipsilateral leg or contralateral leg, or from the leg to the ipsilateral or contralateral arm, is less commonly reported and mainly attributed to trauma. The initial presentation of our patient with vasomotor and hyperesthesia symptoms in the LUE and LLE represents an uncommon pattern in CRPS. While it is atypical for CRPS to affect multiple limbs at onset, our patient’s simultaneous involvement of multiple limbs on the same side of the body further highlights the rarity of this presentation, as supported by the existing literature. In addition to the involvement of the LUE and LLE, our patient’s co-existing cervical radiculopathy and bilateral L5-S1 radiculopathy represent an extreme rarity in the literature. Previous reports focus on bilateral L5-S1 disc protrusions managed conservatively; however, our patient’s complex presentation and failure to respond to medications emphasize the need for a more aggressive approach [9]. This case also highlights the diagnostic challenge of identifying CRPS in the presence of concurrent radiculopathy, as the overlapping features of both conditions can obscure or mimic one another.

Although uncommon, brachial plexopathy is a potential complication of radiation therapy for lung adenocarcinoma [10]. RIBP and CRPS may present with overlapping symptoms, including throbbing pain, extremity swelling, and abnormal sensory changes. Given the patient’s history of thoracic radiation therapy and subsequent development of left deltoid weakness, RIBP was considered as part of the differential diagnosis. However, the absence of detailed radiation treatment records, plexus-targeted electrodiagnostic studies, and dedicated imaging prevents a definitive diagnosis. The clinical findings can also be explained by C5-C7 radiculopathy, which was supported by EMG/NCS findings. Therefore, the possibility of radiation-induced plexopathy is acknowledged but remains unproven and should be interpreted with caution.

This case is notable for its rare presentation of simultaneous CRPS involving the left upper and lower extremities, a pattern that is uncommon compared to most CRPS cases, along with coexisting cervical and lumbar radiculopathy and possible RIBP. The overlapping symptoms of neuropathy, radiculopathy, and CRPS created a significant diagnostic challenge. This case underscores the importance of clinical vigilance and the application of structured diagnostic criteria, such as the Budapest Criteria, to accurately identify CRPS even in the presence of concurrent neurological conditions. It serves as an educational example of how to navigate complex, overlapping pathologies in chronic pain syndromes.

## Conclusions

The overlapping clinical presentation in this case reflects the complexity of diagnosing neuropathic pain syndromes in the setting of multiple neurologic insults. The diagnosis of CRPS Type I was supported by the patient’s clinical history, examination findings, and fulfillment of the Budapest Criteria. Cervical and lumbar radiculopathies were confirmed through both clinical examination and EMG/NCS studies. In contrast, radiation-induced brachial plexopathy remains a suspected but unproven diagnosis, given the lack of dedicated plexus imaging, plexus-specific EMG, or complete radiation treatment records.

The temporal relationship between radiation exposure and the onset of left deltoid weakness raises suspicion but does not establish causation. This diagnostic uncertainty underscores the importance of a systematic, multimodal approach when evaluating overlapping neuropathic conditions. Clinicians should maintain a high index of suspicion for coexisting pathologies and interpret all findings within the broader clinical context.
